# The Influence of Kinesio Tape and an Ankle Brace on the Lower Extremity Joint Motion in Fatigued, Unstable Ankles during a Lateral Drop Landing

**DOI:** 10.3390/ijerph18116081

**Published:** 2021-06-04

**Authors:** Cheng-Chieh Lin, Wan-Chin Lee, Jih-Ching Chen, Shing-Jye Chen, Cheng-Feng Lin

**Affiliations:** 1 Department of Physical Therapy, Graduate Institute of Rehabilitation Science, China Medical University, Taichung 406040, Taiwan; lincc12@mail.cmu.edu.tw; 2 Department of Physical Therapy, College of Medicine, National Cheng Kung University, Tainan 70101, Taiwan; onechie@gmail.com (W.-C.L.); roy565066@gmail.com (J.-C.C.); 3 Department of Product Design, College of Design, Tainan University of Technology, Tainan 71002, Taiwan; te0056@mail.tut.edu.tw; 4 Institute of Allied Health Sciences, College of Medicine, National Cheng Kung University, Tainan 70101, Taiwan; 5 Physical Therapy Center, National Cheng Kung University Hospital, Tainan 70403, Taiwan

**Keywords:** functional ankle instability, external support, kinematics, ankle plantar flexor, landing

## Abstract

Background: An unstable ankle along with plantar flexor muscle fatigue may exacerbate landing performance. External support may be an option to control the ankle motion and protect joints from injuries. Research goal: To investigate the immediate changes in the joint motion of a lower extremity under ankle plantar flexors fatigue conditions in athletes with unstable ankles using different external supports. Methods: A total of 44 participants were allocated to a control (Cn) group, an ankle brace (AB) group, and a kinesio tape (KT) group, and were asked to perform a lateral drop landing before and after a fatigue protocol. The outcome measures were fatigue-induced changes in the maximal joint angle and changes in the angle ranges of the hip, knee, and ankle. Results: Smaller changes in the maximal hip abduction were found in the AB group (*p* = 0.025), and the KT group exhibited smaller changes in the maximal ankle dorsiflexion (*p* = 0.009). The AB group landed with a smaller change in the range of hip flexion and knee flexion (*p* = 0.008 and 0.006). The Cn group had greater fatigue-induced changes in the COM range than AB and KT group (*p* = 0.002 and 0.028). Significance: Despite the beneficial effect in the postural control in the frontal plane, the use of AB might constrain the distal joint motion which might lead to an extended knee landing posture resulting in secondary injuries to the knee joint. Therefore, the use of AB in conjunction with an additional training of landing strategy might be recommended from the injury prevention perspective.

## 1. Introduction

Functional ankle instability (FAI) is a phenomenon in which people have a tendency toward ankle sprains or repeatedly have the feeling the ankle is “giving way” [1]. People with FAI complain of instability in which the joint motion is beyond voluntary control but within physiological limits [2]. According to previous studies, one third of ankle sprains occur during training sessions, and two thirds of ankle sprains occur during matches [3,4,5]. This may suggest that an ankle sprain is related to the continuous muscle contraction of lower extremities during routine training or matches where the athletes go without sufficient rest. The ankle plantar flexor muscles have been proven to play an important role in supporting the body and forward progression since they contribute to the majority of the propulsion force as compared to the knee or hip extensors/flexors from mid-stance phase to toe-off phase [6,7,8]. For athletes, the repetitive jumping or running throughout the game may likely lead to muscle fatigue.

Lower extremity fatigue affects lower body biomechanics and muscle functions in various functional activities [9,10,11,12], and it has negative effects on dynamic stability and leads to injuries such as ankle sprain, slip propensity, and falls [11,12] and alters the loading pattern due to compensatory movement [10]. Thus, fatigue of ankle plantar flexors may prevent athletes from performing tasks that require precise ankle control (i.e., landing).

Previous studies have found that in athletes with FAI, using an external ankle support improves proprioceptive and neuromuscular control and prevents excessive ankle inversion and plantar flexion [13,14,15]. In recent years, Kinesio tape (KT), first introduced by Kenzo Kase, has elastic properties that allows it to stretch up to 140% of its original length, which allows more natural movement than non-elastic adhesive tape. On the other hand, a lace-up ankle brace (AB) is constructed from soft canvas or nylon materials [16]. Although a lace-up AB restricts ankle plantar flexion [17], it increases the immediate muscle reaction induced by a sudden ankle inversion [18] and reduces first-event ankle injuries better than when it is not used [19].

However, Hoch et al. found that the ankle range of motion in people with ankle instability also influences postural control and force attenuation [20,21]. A limited ankle range of motion limits the capacity to transfer impact force through desirable joint motion. Orishimo et al. (2006) found altered control of joint motion in landing after thigh muscle fatigue. They reported a greater demand on the ankle and increased knee flexion responsible for stability [10]. Since ankle sprains usually occur with an unexpected landing before the entire foot contacts the ground, investigations should focus on the immediate ankle reaction from landing to the moment of contacting the ground to determine how external support affects the body’s ability to maintain stability and proper muscle functions. Thus, this study was aimed at evaluating the effects of external support, including AB and KT, on the landing pattern in athletes with ankle instability.

## 2. Materials and Methods

### 2.1. Participants

A total of 33 athletes aged between 18 and 35 years old with functional ankle instability (FAI) were recruited. All participants were screened by a physical therapist and were qualified through Cumberland Ankle Instability Tool (CAIT) developed by Hiller et al. [22]. Inclusion criteria for participation were (1) being a member of the sports team with the frequency of exercise at least 30 min per time and 3 times a week, (2) experiencing at least one significant ankle sprain resulting in pain and swelling, accompanied by the need to rest for a few days in the past two years, (3) experiencing residual symptoms including episodes of giving way, instability, pain, or weakness, (4) scoring <24 on the CAIT along with negative findings on both anterior drawer test and talar tilt test. Participants were excluded from the study if they had swelling or inflammatory symptoms due to an acute ankle sprain, or had a previous fracture, surgery, or congenital bony deficits on the lower extremities, or had any existing neurological disorders or heart disease. All participants read and signed an informed consent form before the study, and the study was approved by the Institutional Review Board of the National Cheng Kung University Hospital (protocol number B-ER-103-198, approved Oct. 2014).

### 2.2. Experimental Procedure

**Figure 1:**
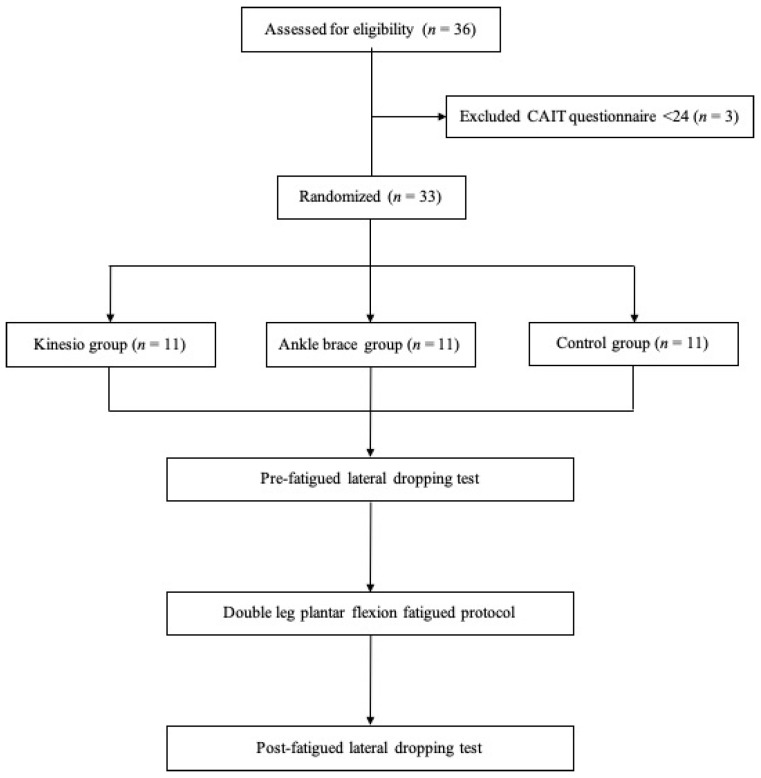
Flow chart of the experimental test procedure for the control (Cn), ankle brace (AB), and kinesio tape (KT) groups.

The participants performed lateral drop landings on the FAI side in the pre-fatigue and post-fatigue condition. Prior to the post-fatigue task, each participant underwent a plantar flexor fatigue protocol (Figure 1). A motion capture system with eight Eagle infrared digital cameras (Motion Analysis Corporation, Santa Rosa, CA, USA) was used to record the real-time 3D trajectory of the reflective markers at a sampling rate of 200 Hz. A total of 42 reflective markers were placed on bony landmarks on the entire body in a modified Helen Hayes marker set, including the top of the head, bilateral front head, sternal notch, 7th cervical spinal process (C7), and the sacrum, as well as bilaterally on the midpoints of the arm, forearm, acromion, lateral and medial epicondyle of the humerus, radial and ulnar styloid process, hand, anterior superior iliac spines (ASIS), midpoint of the thigh and shank, greater trochanters, lateral and medial epicondyle of the knee, and the lateral and medial malleoli.

**Figure 2:**
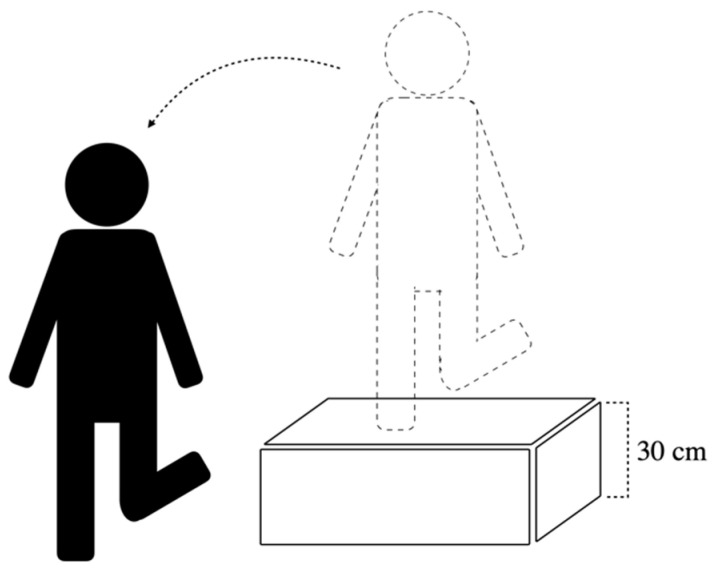
Schematic illustration of the lateral drop landing task.

The participants were instructed to stand on the involved lower limb with the knee extended on a 30 cm-height platform (Figure 2). Maintaining balance for 5 s was required before performing a lateral drop landing on the involved limb on the floor. Participants had to regain stability as soon as possible while keeping the trunk in an upright and forward-facing direction for at least 5 s after landing. Practices and then the rest for several minutes were allowed before the beginning of the trial. Three successful landing trials were collected both before and after the fatigue being induced for each participant. A 30-s rest period was allowed between trials, and the trial was discarded if the participants landed with foot wobbling.

For the purpose of inducing plantar flexor muscle fatigue, participants stood barefoot on a footstool with heels aligned in space with both hands placing slightly forward against the wall to maintain balance. Repetitive maximal ankle plantar flexion was performed without flexing the knees at a rate of 1 time per second (as guided by a metronome). Verbal encouragement and reminder were provided to achieve maximal ankle plantar flexion, and the procedure was not stopped until participants failed to reach 70% of the maximal heel height rise for 3 times consecutively [23]. Borg Rating of Perceived Exertion (RPE), ranging from 6 (no exertion at all) to 20 (maximal exertion) was used to evaluate the subjective feelings of exertion of the participants upon the completion of the fatigue protocol [24].

*External support application.* The AB and KT external support groups wore a lace-up rigid AB (Mueller Sports Medicine, Inc., Prairie Du Sac, USA) and KT (SKT-X-050R, Nitto Denko Corp., Osaka, Japan), respectively, on the involved limb in the fatigue protocol and post-fatigue tasks. For the KT group, the KT was applied on the tibialis anterior, the peroneal longus, and gastrocnemius muscle. The tape was applied from the origin to the insertion of both peroneal longus and tibialis anterior muscle while the ankle was placed in dorsiflexion combined inversion for the former and plantar flexion combined eversion for the latter to ensure consistent muscle facilitation across the participants. An additional tape was applied from the insertion to the origin of the gastrocnemius muscle to prevent muscle over-contraction. Participants were asked to follow the instructions carefully, and the tape was applied on the designated muscle group without tension by the conductor to ensure consistency across all the participants.

### 2.3. Outcome Measures

The outcome measures included in the current study were angles of the hip, knee, and ankle joints and location of center of mass. The joint angle of lower extremity was calculated by means of Eular’s method with the 2-1′-3′′ sequence during motion with X, Y, and Z axis representing the axis of abduction/adduction, flexion/extension, and external/internal rotation, respectively. The whole body center of mass, COM, was calculated by summing up the product of mass distribution in each segment and corresponding COM of segment based on the 14-segment model [25].

### 2.4. Data Analysis

The kinematic data were processed using a self-written algorithm coded in MATLAB (Version R2012b, Mathworks Inc., Natick, MA, USA). The maximal joint angle was measured from landing on the ground to the moment of maximal knee flexion, and the range of the joint angle and COM was obtained by subtracting the maximal range values from the minimal range values. Statistical analyses were performed using SPSS17.0 software (SPSS for Windows, Chicago, IL, USA). In performing the tests, the level of statistical significance was set as *p* < 0.05. Due to the limited number of participants in each group (*N* = 11), the sample size was not normally distributed, and hence, the nonparametric Kruskal–Wallis one-way analysis of variance by ranks test was used. Specifically, the values of all the dependent variables were expressed as a “difference” by subtracting the values obtained in the pre-fatigue task from the values obtained in the post-fatigue task. A mean rank of the “difference” values of the dependent variables in each group was then calculated using the Kruskal–Wallis method in order to determine the significance of the mean rank of the dependent variables among the three groups. A post hoc test was additionally performed to identify group differences.

## 3. Results

Detailed demographic data including CAIT score and duration of exercise were referred to Lin et al. (2020) [26]. The recruited athletes participated in various team sports. The CAIT score ranged from 16~18 in all of the athletes. Thus, the athletes were regarded as having a similar degree of FAI. In the fatigue protocol, the athletes reported *hard* to *extremely hard* degree of with an average RPE score of 18.

### 3.1. Maximal Joint Angle

The AB group (−2.89) had a smaller median value for the difference in hip abduction as compared to the Cn group (−0.11), and a significant group difference (*p* = 0.025) was observed. The post hoc test showed that the fatigue-induced change in the maximal hip abduction for the AB group was significantly smaller than that for the Cn group (*p* = 0.011). The KT group (−5.63) had a smaller median value of difference in ankle dorsiflexion than was the case for the Cn group (1.93), and the post hoc test showed that fatigue-induced changes in the maximal ankle dorsiflexion for the KT group were significantly smaller than that for the Cn group (*p* = 0.009) (Table 1).

### 3.2. Range of Joint Angle

The AB group (−2.45) landed with a smaller median value of difference in hip flexion than the KT group (1.10) (*p* = 0.008). The Cn group (1.94) had greater median value for the difference in hip abduction than the AB group (−1.98) (*p* = 0.006) and the KT group (−1.57) (*p* = 0.045) In terms of the fatigue-induced changes in the knee flexion range, the AB group (−4.68) had a smaller median value than that of the control group (4.98) (*p* = 0.003) and the KT group (1.71) (*p* = 0.014) (Table 2).

### 3.3. The COM Range

The AB group (−0.00316) had a smaller median value for the difference in the COM ML range as compared to the Cn group (0.0047) (*p* = 0.004). In the vertical direction, the Cn group (0.00844) had a greater value for the difference in the COM range as compared to the AB group (−0.00892) (*p* = 0.002) and the KT group (−0.00160) (*p* = 0.028) (Table 3).

## 4. Discussion

This study was aimed toward gaining an understanding of the effects of an external support on the kinematic changes induced by ankle plantar flexor fatigue in athletes with FAI during a lateral drop landing.

After the fatigue protocol, the athletes without external support demonstrated a greater joint angle range and an increased maximal joint angle of the hip and knee in the sagittal and frontal motion while the ankle joint presented greater ankle dorsiflexion. Ankle joint motion has been found to have a relationship with the ground reaction force and has also been found to influence the lower extremity landing pattern in people with ankle instability [21]. Hoch et al. reported that lower dorsiflexion increases the vertical ground reaction force and decreases the ROM at the proximal part of the lower extremities [21]. Rowley et al. reported that a greater contribution of ankle plantar flexion helps to reduce vertical ground reaction force during landing [27]. In the present study, the dorsiflexion angle increased in the Cn group but decreased in both the AB and KT groups after the fatigue protocol, which supports our previous findings that individuals with FAI may have a higher vertical ground reaction force with an AB and KT [26].

The ankle plantar flexor is a key muscle that provides eccentric ankle joint movement, which provides ankle stability during landing in different functional tasks [28,29]. However, the fatigued plantar flexor failed to provide sufficient stability for the ankle joint in our study. The proximal muscles (i.e., gluteus medius, gluteus maximus) have greater muscle strength and volume by which to absorb an impact force as compared to the ankle and thus help with shock absorption [30,31]. However, in the present study the athletes with AB decreased the knee and hip joint angle after fatigue during landing. The possible reason is that the constraint of distal joint motion (i.e., ankle joint) could decrease the range of motion in the proximal joints (i.e., knee joint and hip joint) which may affect the ability of absorbing an impact force for both knee and hip joints [20,21]. The hip and knee joints have been found to attenuate impact at more extended positions by inducing more eccentric contraction and joint stiffness [31,32,33]. However, Simpson et al. reported that people with AB increased the ankle dorsiflexion and peak ground reaction force during landing [34]. This could potentially lead to variance in joint contribution during landing.

Ankle inversion is a risk factor increases the ankle sprain during landing [35,36,37]. Peroneus longus is a key muscle that helps the ankle to stay at eversion in order to prevent excessive ankle inversion [38,39]. One of the main purposes of using KT was to attempt to facilitate the peroneus longus to control frontal motion in the ankle. However, the result was less than we expected. Our findings for the KT group were consistent with previous findings that KT is unable to improve the postural control during a frontal and sagittal plane landing [40]. Additional studies have also shown that KT does not increase muscle activation [41,42] but does increase lymphatic circulation and local blood flow [43]. In addition, KT presented few benefits in increase of ROM and muscle strength [44,45] and the time return to play after musculoskeletal injury [46]. Thus, KT has few positive effects for biomechanics and alter the neuromuscular control.

Center of mass (COM) measurement is commonly used to evaluate the ability of postural control after fatigue [47,48,49]. The athletes without an external support had a greater COM range in the ML and vertical directions during the lateral drop landing, which inferred that fatigue impaired the ability to regulate postural control. Athletes with ankle instability have deficits in their sense of joint position, especially the inversion angle [50,51]. An insufficient preparatory reaction at instantaneous contact after fatigue thereby increases COM postural sway. Furthermore, previous studies have reported that greater COM sway in the frontal plane may result in loss of balance [52,53,54], which has been shown to be a fall indicator in athletes during dynamic control under fatigue conditions. In the present study, the COM in the AB group was decreased in the frontal plane and sagittal plane, and the restricted ankle movement after plantar flexor fatigue indicated that the AB provided additional postural stability during the lateral drop landing even after muscle fatigue. However, one meta-analysis showed that restricted ankle dorsiflexion during landing decreases the proximal joint range of motion [55]. Reduced dorsiflexion may limit the force transition from the hind-foot to the forefoot [56,57], and thus may lower the COM during landing [58]. On the other hand, inconsistent findings have been obtained regarding the effect of KT on the postural control. In our study, there was no significant difference in KT group compared to other two groups in the COM variable while a significantly smaller COP range in KT group in AP direction compared to Cn group and in ML direction compared to Cn and AB groups were reported in Lin et al. (2020) [27]. The above discrepancy indicates that both COM and COP variables should be included to have better understanding on the balance control of the lateral drop landing task.

There are some limitations to the present study. First, there were three times more in number of male than female participants regardless of the similar gender ratio between groups, resulting in the limitation in generalizability of current results. Second, the taping technique was kept consistent across the participants in the present study; however, different muscular structures may be affected due to variation in injury mechanisms of the sports. Therefore, it remains to be investigated whether a different taping technique could achieve better outcome for a specific sport. Third, participants were tested after wearing the external support such as the AB or KT for only several hours; hence, it is unclear whether better effect could be achieved with longer use of external support.

## 5. Conclusions

Our results indicated that fatigue altered the landing strategy into a greater flexion pattern in the lower extremity joints without external support. In athletes with the AB, there was less of a challenge in terms of frontal postural control. However, they would land with a more stiff position and increase injuries in the lower extremities. The application of external ankle support may enhance posture control but may potentially increase the risk of injury in the knee joint during landing. It is concluded from current findings that the use of KT might not be sufficient to result in the alteration of biomechanics of lower extremity joints during landing, and the combination of the training on landing strategy and external ankle support may be needed to achieve the purpose of ankle protection without compromising other joints of the lower extremities during landing for individuals with unstable ankles.

## Figures and Tables

**Table 1:** Kruskal–Wallis and post hoc test results for fatigue-induced changes in the maximal joint angle in lateral drop landing tasks for the three groups.

	Cn			AB			KT			
	Mean	Median	Mean Rank	Mean	Median	Mean Rank	Mean	Median	Mean Rank	K-W Test
Hip										
Sagittal Flex(+) Ext(-)	2.79	1.25	17.27	1.58	1.60	16.64	3.97	0.46	17.09	0.987
Frontal ABD(+) ADD(-)	0.72	−0.11	23.18	−3.26	−2.89 ^a^	12.27	−2.47	−1.67	15.55	0.025 *
Transverse ER(+) IR(-)	0.09	−0.27	18.45	−1.67	0.29	17.36	−1.69	−1.86	15.18	0.721
Knee										
Sagittal Flex(+) Ext(-)	4.74	3.67	20.73	0.22	−1.55	12.64	3.36	3.02	17.64	0.141
Frontal ABD(+) ADD(-)	0.15	−0.54	19.55	−0.77	0.25	18.64	−4.34	−4.53	12.82	0.209
Transverse ER(+) IR(-)	−3.97	−1.33	16.55	−0.91	0.48	20.82	−4.43	−5.15	13.64	0.215
Ankle										
Sagittal DF(+) PF(-)	2.50	1.93	22.55	−1.68	−3.43	16.73	−6.74	−5.63 ^b^	11.73	0.032 *
Frontal ABD(+) ADD(-)	3.47	−2.82	17.64	−2.58	−3.35	12.18	2.49	1.03	21.18	0.089
Transverse ER(+) IR(-)	4.62	3.78	16.59	3.73	2.91	15.91	5.83	5.45	18.50	0.809
Trunk										
Sagittal Flex(+) Ext(-)	2.76	0.43	17.59	2.45	1.56	17.55	1.63	0.60	15.86	0.892
Frontal Medial(+) Lateral (-)	−0.53	−0.07	19.55	−0.94	−0.55	15.73	−0.66	−0.71	15.73	0.565

*: significant difference; Flex: flexion; Ext: extension; ABD: abduction; ADD: adduction; ER: external rotation; IR: internal rotation; DF: dorsiflexion; PF: plantar flexion; ^a^: A significant smaller difference in the maximal hip ABD in AB group as compared to the Cn group (*p* = 0.011); ^b^: A significant smaller difference in the maximal ankle DF in the KT group as compared to the Cn group (*p* = 0.009).

**Table 2:** Kruskal–Wallis and post hoc test results for fatigue-induced changes in the range of the joint angle in lateral drop landing tasks performed by the three groups.

	Cn			AB			KT			
	Mean	Median	Mean Rank	Mean	Median	Mean Rank	Mean	Median	Mean Rank	K–W Test
Hip										
Sagittal Flex(+) Ext(-)	2.28	3.00	19.73	−1.46	−2.45 ^a^	10.64	2.65	1.10	20.64	0.027 *
Frontal ABD(+) ADD(-)	1.37	1.94	23.55	−2.23	−1.98 ^b^	12.82	−2.86	−1.57 ^c^	14.64	0.021 *
Transverse ER(+) IR(-)	1.38	1.48	19.64	0.81	0.05	17.55	−1.90	−1.64	13.82	0.360
Knee										
Sagittal Flex(+) Ext(-)	4.58	4.98	21.45	−2.57	−4.68 ^d,^^e^	9.55	4.51	1.71	20.00	0.007 *
Frontal ABD(+) ADD(-)	0.65	1.00	19.86	−0.23	−0.03	16.64	−1.43	−0.52	14.50	0.424
Transverse ER(+) IR(-)	0.75	1.13	17.27	0.97	1.28	17.73	0.68	0.53	16.00	0.910
Ankle										
Sagittal DF(+) PF(-)	5.01	5.37	22.55	−0.85	1.94	12.82	1.47	1.76	15.64	0.052
Frontal ABD(+) ADD(-)	3.23	2.34	21.00	−1.06	−0.79	13.64	0.78	.01	16.36	0.196
Transverse ER(+) IR(-)	1.61	0.20	22.00	−1.34	2.01	17.00	−4.14	−3.59	12.00	0.053
Trunk										
Sagittal Flex(+) Ext(-)	1.69	1.64	20.86	0.40	0.46	15.50	0.32	−0.35	14.64	0.262
Frontal Medial(+) Lateral (-)	0.85	0.82	20.86	0.20	0.23	15.50	0.16	−0.17	14.64	0.262

*: significant difference; Flex: flexion; Ext: extension; ABD: abduction; ADD: adduction; ER: external rotation; IR: internal rotation; DF: dorsiflexion; PF: plantar flexion; ^a^: A significant smaller difference in the range of hip flexion in the AB group as compared to the KT group (*p* = 0.008); ^b^: A significant smaller difference in the range of the hip ABD in the AB group as compared to the Cn group (*p* = 0.006); ^c^: A significant smaller difference in the range of the hip ABD in the KT group as compared to the Cn group (*p* = 0.045); ^d^: A significant smaller difference in the range of knee flexion in the AB group as compared to the Cn group (*p* = 0.003); ^e^: A significant smaller difference in the range of knee flexion in the AB group as compared to the KT group (*p* = 0.014).

**Table 3:** Kruskal–Wallis and post hoc test results for fatigue-induced changes in the COM range in lateral drop landing tasks performed by the three groups.

	Cn			AB			KT			
	Mean	Median	Mean Rank	Mean	Median	Mean Rank	Mean	Median	Mean Rank	K–W Test
Difference of COM range (%BH)										
ML	0.00507	0.00470	23.45	−0.00332	−0.00316 ^a^	11.09	0.00014	−0.00094	16.45	0.011 *
AP	−0.00064	0.00059	19.36	−0.00254	−0.00264	14.45	−0.00067	−0.00159	17.18	0.491
Vertical	0.00788	0.00844	24.27	−0.00832	−0.00892 ^b^	11.36	−0.00433	−0.00160 ^c^	15.36	0.006 *

*: significant difference; ML: medial–lateral; AP: anterior–posterior; BH: body height; ^a^: A significant smaller difference in the COM ML range in the AB group as compared to the control group (*p* = 0.004); ^b^: A significant smaller difference in the COM vertical range in the AB group as compared to the control group (*p* = 0.002); ^c^: A significant smaller difference in the COM vertical range in the KT group as compared to the control group (*p* = 0.028).

## Data Availability

The data that support the findings of this study are available from the corresponding author upon reasonable request.
